# Fault Feature Extraction of Rolling Bearings Based on Ordered Singular Spectrum Decomposition—Multipoint Optimal Minimum Entropy Deconvolution Adjusted

**DOI:** 10.3390/e28070797

**Published:** 2026-07-14

**Authors:** Longlong Li, Wenhao Chen, Wenhui Li, Yan Zhang, Jiaxin Liu, Runlin Chen

**Affiliations:** 1 School of Mechanical Engineering, Shaanxi Polytechnic University, Xianyang 712000, China; 20191499@sxpi.edu.cn; 2 School of Mechanical Engineering, Xi’an University of Technology, Xi’an 710048, China; 2240221259@stu.xaut.edu.cn (W.C.); 2240221192@stu.xaut.edu.cn (W.L.); xut2210221225@163.com (J.L.); chenrunlin@xaut.edu.cn (R.C.)

**Keywords:** Ordered Singular Spectrum Decomposition, Multipoint Optimal Minimum Entropy Deconvolution Adjusted, early fault detection, rolling bearings

## Abstract

In the early fault stage of rolling bearings, the fault-induced impact signals in vibration data are often extremely weak and easily obscured by strong noise, making effective extraction and analysis challenging. To address this issue, this paper proposes a novel weak fault impact signal feature extraction method combining Ordered Singular Spectrum Decomposition (OSSD) and Multipoint Optimal Minimum Entropy Deconvolution Adjusted (MOMEDA). First, OSSD is employed to decompose the raw vibration signal, progressively extracting signal components across different frequency bands. The optimal signal components are adaptively selected based on mutual information criteria, effectively avoiding mode mixing issues. Subsequently, MOMEDA is applied to enhance the periodic impact features within the fault signal, improving its recognizability. To address the signal length reduction issue inherent in the MOMEDA process, a waveform extension strategy is introduced to compensate for the missing signal, ensuring signal integrity. Simulation and experimental results demonstrate that the proposed method exhibits robust noise resistance and can effectively extract early fault features of rolling bearings under strong noise conditions, validating its accuracy and effectiveness.

## 1. Introduction

Rolling element bearings are critical components in rotating machinery, and their operating condition directly affects equipment safety and reliability [1]. This is particularly important in high-speed and high-precision systems such as machine tool electric spindles, where bearing performance plays a decisive role in machining quality and operational stability [2]. In recent years, various methods have been proposed to address bearing fault diagnosis under complex operating conditions [3].

Che et al. [4] proposed an improved domain adaptation-based fault diagnosis method, which achieves cross-domain feature alignment through multi-layer multi-kernel maximum mean discrepancy and adversarial learning, thereby enhancing the model’s generalization capability under different working conditions. Zheng et al. [5] proposed a generalized refined composite multiscale fuzzy entropy (GRCMFE) to improve the stability and accuracy of complexity measurement. By integrating multi-cluster feature selection and a support vector machine optimized by the gravitational search algorithm, an intelligent fault diagnosis framework was developed. Wu et al. [6] developed a diagnostic framework combining refined multiscale rating entropy with an optimized extreme learning machine to address the difficulty of incipient fault detection under small-sample conditions, effectively improving diagnostic stability and accuracy. Cheng Junsheng et al. [7] applied the empirical mode decomposition (EMD) method to analyze vibration signals from rolling bearings. This method decomposed the vibration signal into multiple intrinsic mode function (IMF) components and constructed an initial feature vector matrix based on these components. Wang Kun et al. [8] addressed the issue of insufficient fault samples in intelligent monitoring and fault diagnosis by using empirical mode decomposition for data preprocessing. They extracted energy features at different frequency bands as input parameters for support vector data description (SVDD) classification. Experimental results showed that this method could more effectively identify the operating state of bearings compared to traditional SVDD methods. Zheng Xiaoxia et al. [9] improved the traditional EMD method using differential techniques to solve wave mixing problems in rolling bearing fault diagnosis. By increasing the energy ratio of high-frequency components, they achieved better extraction of fault components. Wang Wenbo et al. [10] proposed a fault diagnosis method combining adaptive fusion of time-varying filtering-EMD modal components and singular value decomposition denoising to address difficulties in extracting early weak fault features under complex operating conditions. Lu Zhijie et al. [11] tackled Variational Modal Decomposition (VMD) parameter optimization issues by proposing a new solution approach focusing on fitness function construction and improvements in swarm intelligence algorithms. They also explored the inadequacies of VMD in diagnosing early weak faults and compound faults in rolling bearings. Yu Jun et al. [12] combined the Chimp Optimization Algorithm (ChOA) with VMD to propose an adaptive VMD algorithm based on ChOA. They selected effective modal components for reconstruction to reduce interference from strong background noise. Gu Jiefei et al. [13] used the VMD method to select optimal modal components from fault signals and then enhanced the shock components in these optimal components using Maximum Correlated Kurtosis Deconvolution. Finally, envelope spectrum analysis was employed to extract fault frequencies, effectively identifying rolling bearing fault characteristics submerged in strong noise. Ma Zhenrong et al. [14] used the RIME algorithm to determine the optimal combination of decomposition components and penalty factors in VMD. They calculated the kurtosis values of each decomposed IMF component and selected the most prominent fault feature components for reconstruction and denoising. The sample entropy of the reconstructed signal was then computed as a fault feature and fed into a support vector machine for rapid identification and diagnosis of various types of rolling bearing faults. Wang Xingbing et al. [15] addressed the low diagnostic accuracy and long processing times associated with traditional models due to neglect of data correlation and inability to effectively handle nonlinear signals. They preprocessed one-dimensional vibration signals using ensemble empirical mode decomposition (EEMD). Huang Xiaoxiao et al. [16] proposed a method combining EEMD with multi-parameter constrained time-delay feedback tri-stable stochastic resonance (MCTFTSR) systems to diagnose fault signals characterized by non-stationarity, multi-components, and multiple interferences. They conducted comparative analyses with EEMD and MCTFTSR methods. Damine Yasser et al. [17] developed an EEMD-based bearing fault diagnosis method to overcome the problem of rich noise in vibration signals when bearings have faults, making it difficult to obtain information about the bearing’s condition from the signal. They used the three-sigma rule for denoising to enhance periodic impulses. Damine Yasser [18] addressed the IMF selection problem in EEMD for bearing fault diagnosis by proposing a combined modal ensemble empirical mode decomposition method to directly obtain effective IMF combinations containing fault information. Zhen Dong et al. [19] proposed a fault feature extraction method based on improved EEMD and modulated signal bispectrum to improve the signal-to-noise ratio and suppress random noise interference, considering the strong nonlinearity and non-stationarity of rolling bearing vibration signals. Dou ChunHong et al. [20] introduced Singular Spectrum Decomposition (SSD) to overcome mode mixing problems in EMD and EEMD for rolling bearing fault signals’ non-stationary and nonlinear characteristics, proposing an SSD-based bearing fault feature enhancement method. Xu Weiyang et al. [21] proposed an improved SSD algorithm based on permutation entropy to adaptively determine the number of SSD layers and enhance the ability to extract weak fault features from noisy signals. Pang Bin et al. [22] proposed an enhanced SSD method to overcome the inability of SSD to effectively separate fault signals with less prominent frequencies, enhancing fault detection capability through the introduction of differential and integral operators. Wang Shenquan et al. [23] proposed a fault diagnosis method based on SSD and optimized stochastic configuration networks to improve recognition capabilities for complex signals in challenging environments where weak fault signals in rolling bearings are overwhelmed by interference. Jiang Lingli et al. [24] proposed a lightweight convolutional neural network (CNN) model combined with network pruning algorithms and neural architecture search to optimize model structure, addressing issues of high computational resource requirements and storage overheads in deep CNNs for rolling bearing fault diagnosis. Yuan Jianhu et al. [25] proposed an intelligent fault diagnosis method based on wavelet time–frequency representations and CNNs to enhance feature extraction and recognition capabilities, optimizing the CNN model after continuous wavelet transform for improved classification accuracy. Iu et al. [26] proposed a fault diagnosis method that combines the Markov Transition Field and CNN. In this approach, the Markov Transition Field is first used to transform one-dimensional time series signals into two-dimensional image representations, and then the CNN automatically extracts deep features to achieve fault classification. Gu Kai et al. [27] proposed a multi-sensor fault diagnosis method combining discrete wavelet transform (DWT) and long short-term memory networks (LSTMs) to accurately diagnose rolling bearing fault states, extracting detailed fault information with DWT and learning long-term dependencies in time series with LSTMs. Taibi Ahmed et al. [28] proposed a bearing induction fault diagnosis method combining VMD, DWT, composite multiscale weighted permutation entropy, and locally sensitive discriminant analysis to improve fault feature extraction and classification capabilities, addressing the impact of bearing faults on the operation of induction motors.

Currently, there is still a lack of effective methods for extracting early fault features in rolling bearings, especially in terms of detecting and recognizing weak fault signals. To address these challenges, this paper proposes a fault feature extraction method combining OSSD with MOMEDA. Through verification using simulation signals and experimental data, the proposed method can effectively extract weak fault features in rolling bearings, improving the accuracy and reliability of fault diagnosis. Compared with existing research, the waveform extension preprocessing addresses the feature dispersion problem of weak bearing fault signals under strong noise interference. It is well-adapted to the OSSD-MOMEDA model for early weak faults in rolling bearings, which helps to improve the performance of early weak fault extraction under low-signal-to-noise-ratio conditions.

## 2. Fault Feature Extraction Principles

### 2.1. Singular Spectrum Decomposition

In the early fault detection of rolling bearings, traditional time–frequency analysis methods such as EMD and VMD often face challenges like mode mixing and endpoint effects in practical applications, making it difficult to effectively extract weak fault characteristic signals. Singular Spectrum Decomposition [29] can effectively decompose non-stationary signals and avoid mode mixing. The specific process is as follows:

Trajectory Matrix Construction: For a time series t, with a data length of *N* and an embedding dimension *M*, take the sequence x(i)=[a,b,c,d,e] as an example. When the embedding dimension *M* is 3, the embedded matrix *X* is as shown in Equation (1).

(1) $$X=\left[\begin{array}{ccc} a & b & c\\ b & c & d\\ c & d & e \end{array}\begin{array}{cc} d & e\\ e & a\\ a & b \end{array}\right]$$

In matrix *X*, the *i* row is $x_{i}=(x(i),\ldots ,\ldots x(N),x(1),\ldots x(i-1))$, where i=1,2,3…,M. The portion on the left side, delineated by a dashed line, represents the trajectory matrix. To enhance the oscillatory components of the signal, shift the elements $\left[\begin{array}{cc} & a\\ a & b \end{array}\right]$ on the right side of matrix *X* to the upper left, resulting in a new matrix as shown in Equation (2):

(2) $$a\;=\;1,\;X=\left[\begin{array}{ccc} & & a\\ & a & b\\ a & b & c\\ b & c & d\\ c & d & e \end{array}\begin{array}{cc} d & e\\ e & \end{array}\right]$$

In the equation, the dashed section represents the trajectory matrix in the new singular value decomposition, where the diagonal elements are identical and equal in quantity.

Adaptive determination of embedding dimension *M*: An adaptive strategy is used to select the embedding dimension *M* in the *j*-th iteration. Therefore, the power spectral density of the residual component in the *j*-th iteration is

(3) $$u_{j}(n)=x(n)-\sum_{k=1}^{j-1}u_{k}(n)a=1,$$

(4) $$u_{0}(n)=x(n)$$

If the maximum peak frequency *f*_max_ does not reach the set threshold (10^−3^), the residual is treated as the main trend component. After that, the residual is recalculated in each iteration. After the first iteration, $M=1.2(F_{s}/f_{\max})$.

Reconstruction of the *j*-th SSC component: If only a single significant trend component is identified during the first iteration, the first left and right eigenvectors are used to obtain $g^{\left(1\right)}(n)$. In subsequent iterations, the frequency of component $g^{\left(1\right)}(n)$ is mainly distributed within $\left[f_{\max}-\delta_{f},f_{\max}+\delta_{f}\right]$, where $\delta_{f}$ is half of the main peak bandwidth of the main density of the residual signal power spectrum of $u_{j}(n)$. The main frequency and the portion with the most significant peak energy are grouped together to form set $I_{j}=[i_{1},i_{2},\ldots i_{q}]$, which is then used to reconstruct the SSC component.

Iteration termination: The SSC component ${\tilde{g}}^{\left(j\right)}(n)$ from each iteration is separated, and the normalized mean square error (*NMSE*) between the residual signal and the original signal is evaluated:

(5) $$NMSE=\frac{\sum_{i=1}^{N}\left(u^{\left(j+1\right)}{\left(i\right)}^{2}\right)}{\sum_{i=1}^{N}{\left(x(i)\right)}^{2}}$$

When the *NMSE* is lower than the preset threshold, the iteration stops; otherwise, the residual signal replaces the original signal, and the iteration continues until the condition is met. The final decomposition result is as shown in Equation (6):

(6) $$x(n)=\sum_{k=1}^{m}{\tilde{g}}^{\left(k\right)}(n)+u^{\left(m+1\right)}(n)$$

where *m* is the number of obtained SSC components.

### 2.2. Ordered Singular Spectrum Decomposition

After the original signal is decomposed using SSD, SSC components are obtained by dividing them into different frequency bands. Effective decomposition can better suppress the modal aliasing problem, so setting the number of components is very important. The traditional SSD algorithm’s iteration termination method does not consider the evolution rule of the signal itself, making it less adaptable to nonlinear, non-stationary fault signals. In OSSD, the number of components is crucial. Choosing the appropriate value of *K* can avoid losing important characteristic information due to selecting too few components or generating spurious components due to selecting too many. This selection can be evaluated using the mutual information criterion to ensure that the chosen components can represent the main features of the signal.

Mutual information [30] analyzes the amount of shared information between random variables. The mutual information between two random variables *X* and *Y* is given by (7):

(7) $$I(X;Y)=\sum_{x}\sum_{y}p(x,y)\log\frac{p(x,y)}{p(x)p(y)}$$

**Figure 1:**
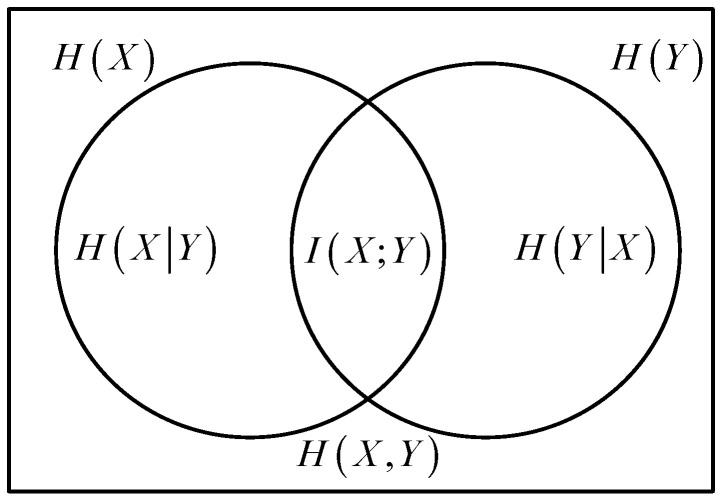
Mutual information.

The probability density functions of *p*(*x*), *p*(*y*) and *p*(*x*, *y*) are defined as shown in Figure 1.

### 2.3. Multipoint Optimal Minimum Entropy Deconvolution

#### 2.3.1. Principle of Momeda

After OSSD, although weak fault signals have been effectively separated, some impact features are still masked by noise. Therefore, to further enhance the impact features of the fault signal and effectively extract fault information, MOMEDA is introduced.

Let the impact signal caused by bearing faults be y, *n* be the noise signal, and *h* be the frequency response function of the vibration system. Thus, the time-domain signal collected by the vibration sensor is given by Equation (8):

(8) x=h×y+n

Starting from the bandpass theory, MOMEDA aims to find the optimal FIR filter to reconstruct the original impact signal while reducing the influence of noise on the impact signal. Therefore, its convolution process is given by Equation (9):

(9) $$x=h\times s=\sum_{k=1}^{N-1}f_{k}s_{k+L-1}\;\;\;\;k=1,2,\ldots ,N-L$$

where *N* is the number of sampling points, and *L* is the filter length.

The vibration signal of a rolling bearing fault exhibits periodic vibration impacts. Based on the D-norm, MOMEDA introduces the multipoint D-norm to achieve the maximum reconstruction of the signal, as shown in Equations (10) and (11).

(10) $$MDN(y,t)=\frac{1}{\left\|t\right\|}\frac{t_{T}y}{\left\|y\right\|}$$

(11) $$MOMEDA=\underset{f}{\max}MDN(y,t)=\underset{f}{\max}\frac{t\cdot y}{\left\|y\right\|}$$

In these equations, MDN represents the multipoint D-norm, and MOMEDA is the maximum multipoint norm. The target vector is *t*, which defines the position and weight of the target impact component.

To obtain the optimal FIR filter, the correlation coefficient $\left[f_{1},f_{2},\cdots ,f_{L}\right]$ of the filter is used to solve Equation (11), resulting in Equation (12).

(12) $$\frac{d}{df}\left(\frac{t^{T}y}{\left\|y\right\|}\right)=\frac{d}{df}\frac{t_{1}y_{1}}{\left\|y\right\|}+\frac{d}{df}\frac{t_{2}y_{2}}{\left\|y\right\|}+\ldots +\frac{d}{df}\frac{t_{N-L}y_{N-L}}{\left\|y\right\|}$$

Since

(13) $$\frac{d}{df}\left(\frac{t^{T}y}{\left\|y\right\|}\right)={\left\|y\right\|}^{-1}t_{k}M_{k}-{\left\|y\right\|}^{-3}t_{k}y_{k}X_{0}y$$

And

(14) $$M_{k}={\left[x_{k+L-1},x_{k+L-2},\ldots x_{k}\right]}^{T}$$

Equation (13) can be represented as

(15) $$\begin{array}{l} \frac{d}{df}\left(\frac{t^{T}y}{\left\|y\right\|}\right)={\left\|y\right\|}^{-1}(t_{1}M_{1}+t_{2}M_{2}+\ldots +t_{k}M_{k})\\ -{\left\|y\right\|}^{-3}t^{T}yX_{0}y \end{array}$$

Simplifying Equation (15):

(16) $$t_{1}M_{1}+t_{2}M_{3}+\ldots t_{N-L}M_{N-L}=X_{0}t$$

When its derivative is 0, Equation (16) becomes

(17) $${\left\|y\right\|}^{-1}X_{0}t-{\left\|y\right\|}^{-3}t^{T}yX_{0}y=0$$

Since $y=X_{0}^{T}f$, and when ${\left(X_{0}X_{0}^{T}\right)}^{-1}$ exists, it can be expressed as

(18) $$\frac{t^{T}y}{{\left\|y\right\|}^{2}}f={\left(X_{0}X_{0}^{T}\right)}^{-1}X_{0}t$$

Thus, the multiple of the filter $f={\left(X_{0}X_{0}^{T}\right)}^{-1}X_{0}t$ is the solution to MOMEDA, where *X*_0_ is given by Equation (19):

(19) $$X_{0}={\left|\begin{array}{cccc} x_{L} & x_{L+1} & \ldots & x_{N}\\ x_{L-1} & x_{L} & \ldots & x_{N-1}\\ \ldots & \ldots & \ldots & \ldots \\ x_{1} & x_{2} & \ldots & x_{N-L+1} \end{array}\right|}_{L\times \left(N-L+1\right)}$$

#### 2.3.2. Waveform Extension

When using MOMEDA to enhance fault signal features, the deconvolution operation reduces the length of the resulting signal. To minimize the loss of fault signal, a signal mirroring extension process is adopted, as follows:

Identify the local extreme points of the convolved signal and their positions.

**Figure 2:**
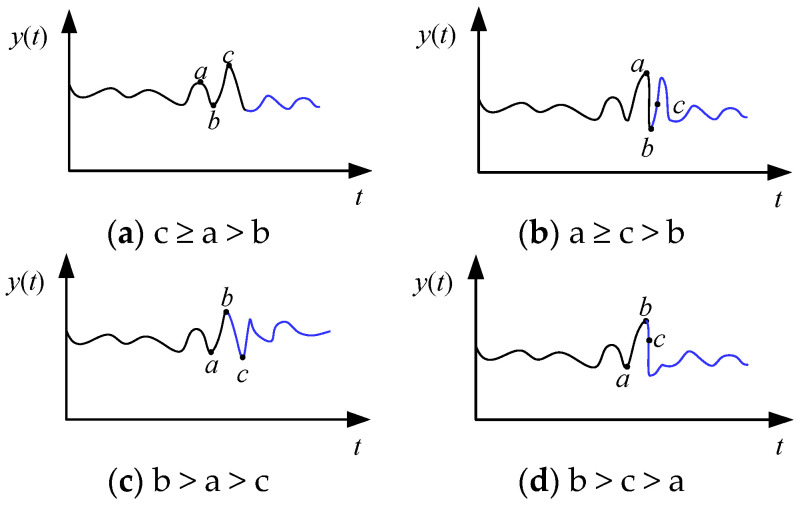
Waveform extension under different conditions.

Determine the center of the waveform extension based on the local features of the signal at the boundaries. Let *a*, *b*, *c* be the local maximum or minimum point of the signal. As shown in Figure 2a, when c≥a>b, *c* is the symmetry point. As shown in Figure 2b, when a≥c>b, *a* is the symmetry point. As shown in Figure 2c, when b>a≥c, *b* is the symmetry point. As shown in Figure 2d, when b>c≥b, *b* is the symmetry point.

The data before the symmetry point is selected as the extension object, and a mirror extension is performed with the symmetry point as the center. The extension result is shown as the blue solid line in Figure 2.

### 2.4. Fault Feature Extraction Process

**Figure 3:**
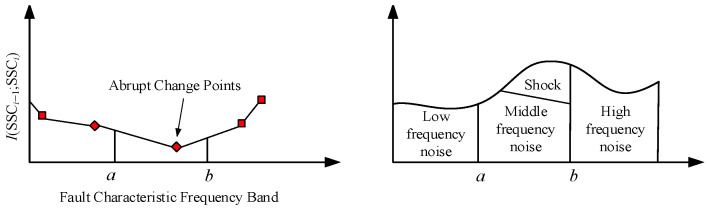
Determination of frequency band of fault signal.

**Figure 4:**
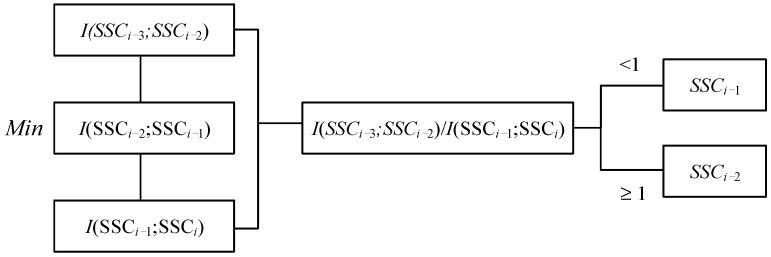
Determination of the best component.

**Figure 5:**
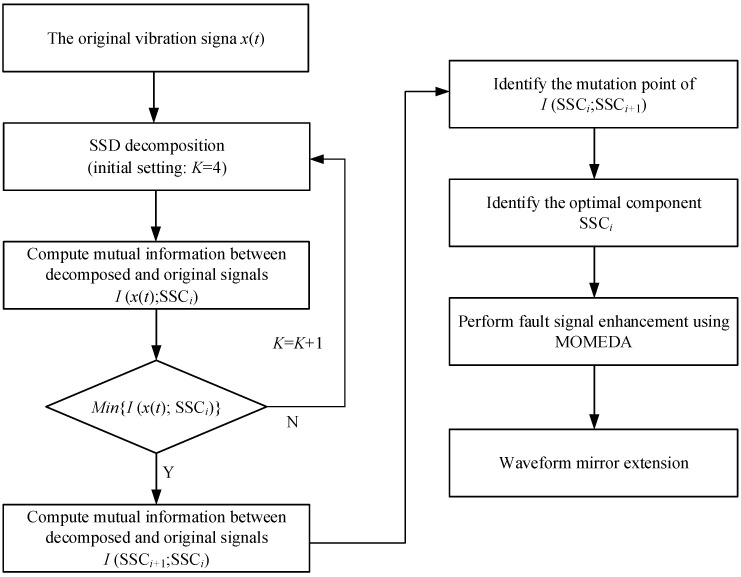
The flow chart of OSSD-MOMEDA.

(1) The original vibration signal is adaptively decomposed using OSSD to obtain SSC components across different frequency bands.
(2) The mutual information between adjacent components is calculated to identify abrupt change points in the curve and determine the fault characteristic frequency band, as shown in Figure 3.
(3) The mutual information between each component within the frequency band and the original signal is compared to select the optimal SSC component containing fault features, as shown in Figure 4.
(4) The optimal component is enhanced using MOMEDA, and a signal mirror extension strategy is employed to eliminate endpoint effects and restore the signal to its original length. The detailed procedure is illustrated in Figure 5.

## 3. Simulation Signal Verification

### 3.1. Simulation Signal Construction

To verify the feasibility of the method proposed in this paper, a simulation signal *x*(*t*) for the inner race fault of a rolling bearing is constructed as shown in Equation (20). The sampling frequency is 16,000 Hz, and the number of sampling points is 4096.

(20) $$\left\{\begin{array}{l} x(t)=s(t)+n(t)=\sum_{i}A_{i}h(t-iT)+n(t)\\ h(t)=\exp(-Ct)\cos(2\pi f_{n}t)\\ A_{i}=1+A_{0}\cos(2\pi f_{r}t) \end{array}\right.$$

In the formula, s(t) is the periodic shock signal caused by the inner ring failure, $A_{0}$ is the amplitude of the periodic shock signal, $f_{r}$ is the rotational frequency of the rolling bearing, C is the attenuation coefficient, $f_{n}$ is the resonance frequency, $f_{i}$ is the characteristic frequency of the inner ring failure, n(t) is the noise signal, and h(t) is the exponential decay signal. The specific simulation parameters are illustrated in Table 1.

### 3.2. Signal Decomposition and Feature Extraction

#### 3.2.1. OSSD

**Figure 6:**
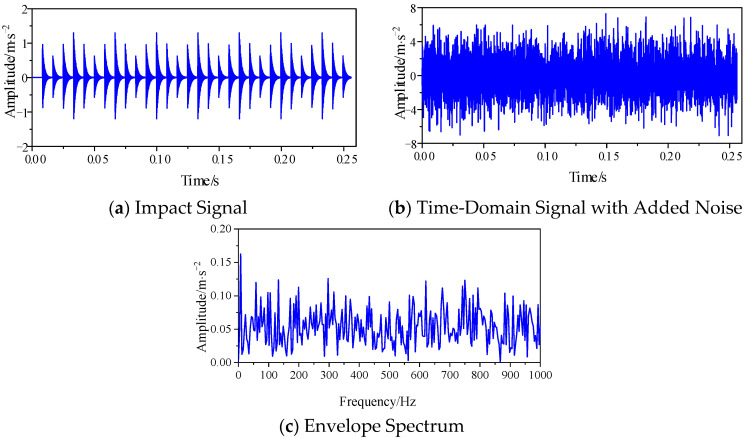
Time domain and frequency domain of simulation signal.

The simulated impact signal, inner race fault signal of the rolling bearing, and their envelope spectra are shown in Figure 6. Under the influence of noise signals, the periodic impact signal is completely submerged, making it difficult to identify prominent frequency components after envelope spectrum analysis.

**Figure 7:**
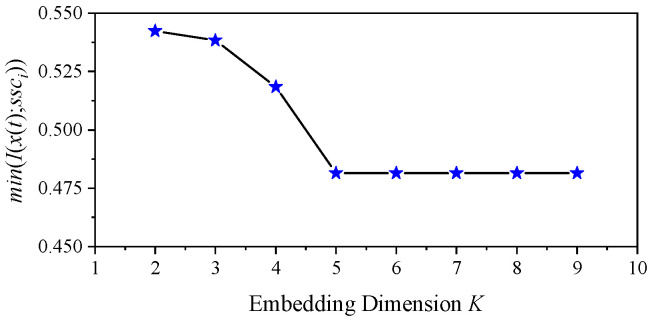
Determine the optimal embedding dimension *K* for the simulation signal.

The method proposed in this paper is used to analyze the simulated signal, and the calculation results are shown in Figure 7. The optimal embedding dimension is determined as 5 by calculating the mutual information between the decomposed signal components and the original signal.

**Figure 8:**
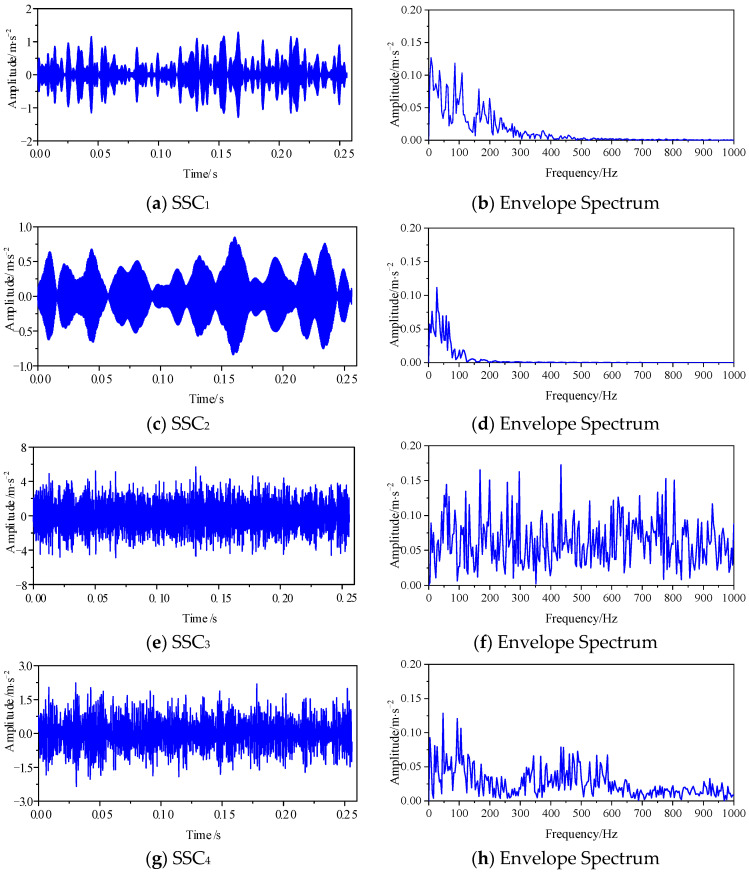

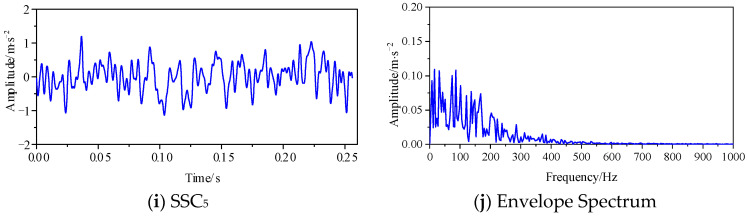
The signal after OSSD and its envelope spectrum.

The time-domain signals and spectral plots of the fault signal after OSSD are shown in Figure 8.

**Figure 9:**
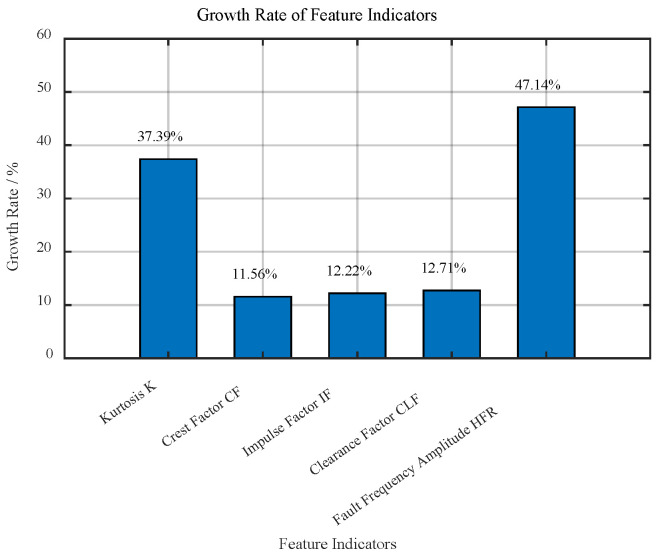
Improvement rates of various indicators for the SSC3 component compared to the noise-added signal before decomposition.

The quantitative analysis results are shown in Figure 9 and Table 2. After SSD, the various impact feature indicators of the third component are all improved compared to those before decomposition. Notably, the fault harmonic energy ratio in the envelope spectrum increases by approximately 47.14%, indicating that this component has a more significant enhancing effect on the periodic fault-induced impact components.

#### 3.2.2. Selection of Optimal Components

**Figure 10:**
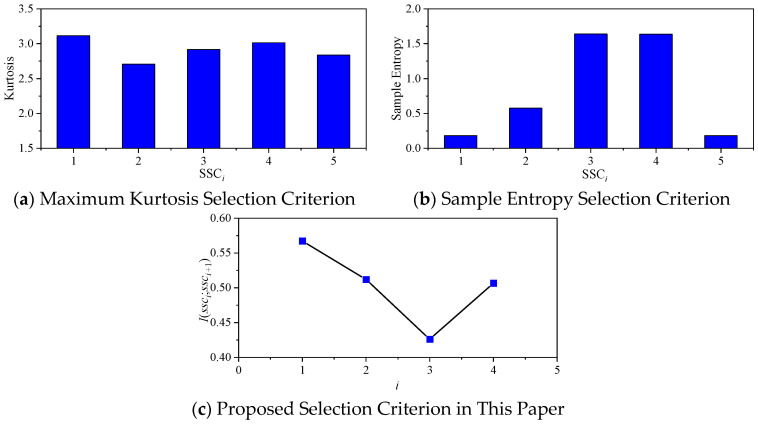
Different selection criteria.

**Figure 11:**
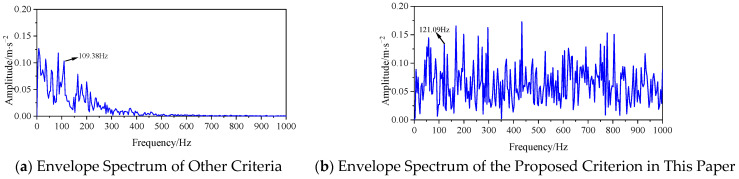
Envelope spectra of different criteria.

To determine the optimal component, traditional kurtosis criteria, sample entropy criteria, and the selection criterion proposed in this paper are used, as shown in Figure 10:

Using traditional kurtosis and sample entropy criteria, the kurtosis value of SSC_1_ is the largest and the sample entropy is the smallest, so it is selected as the optimal component, and its envelope spectrum analysis is performed. The result is shown in Figure 11.

From Figure 11a, it can be seen that the prominent frequency of the envelope spectrum is 109.38 Hz, with an error of 8.85% compared to the theoretical value of 120 Hz. Only part of the frequency bands are present, and the signal’s harmonics are not fully represented. This indicates that under strong noise conditions, traditional kurtosis and sample entropy criteria are susceptible to impulsive interference, leading to misjudgments in component selection and exhibiting poor adaptability to early weak faults [31]. When using the selection criterion proposed in this paper, a mutation point appears at *i* = 3, and the optimal components lie between SSC_3_ and SSC_4_. The results for *i* = 2 and *i* = 4 are compared, and the optimal component is determined to be SSC_3_. The envelope spectrum analysis of SSC_3_ is shown in Figure 11b, where the prominent frequency peak is 121.09 Hz, with only a 0.91% error compared to the theoretical value of 120 Hz, but other harmonics are not prominent. Therefore, processing the vibration signal with OSSD alone does not make the fault characteristics significant. To enhance the fault features, MOMEDA is applied to SSC_3_.

### 3.3. Momeda Signal Processing and Signal Extension

**Figure 12:**
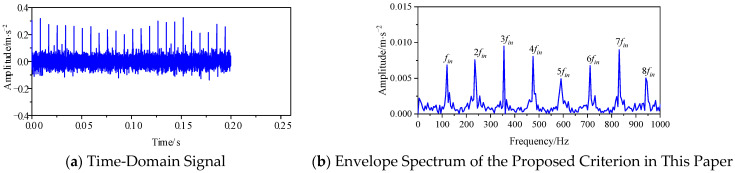
MOMEDA signal processing of the simulation signal.

The selected optimal component is processed using MOMEDA. Based on the fault frequency identified in Figure 11b, the convolution period is set to 133 by utilizing the mapping relationship between period and frequency. In the MOMEDA algorithm, the selection of the filter length L is crucial and generally needs to satisfy the constraint of 2T to 5T [32]. To ensure sufficient frequency-domain resolution while controlling computational overhead and avoiding noise overfitting, as well as to accommodate the uniform coverage requirements for different fault periods in subsequent practical experiments, the filter length is set to 900 for the simulation signal and 600 for the experimental signals in this study. Figure 12 shows the time-domain signal and envelope spectrum after MOMEDA processing.

**Figure 13:**
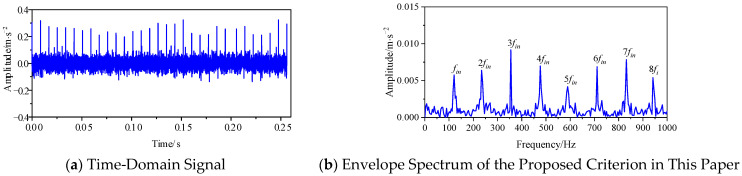
The time domain and envelope spectrum of the signal after waveform extension.

As shown in Figure 12, the inner race fault characteristic frequency and its harmonics are clearly visible, indicating that the rolling bearing has an inner race fault, which is consistent with expectations. However, the time-domain signal after processing shows some missing data. To reduce signal loss, a signal mirroring extension strategy is used to restore the signal to its original length. Figure 13 shows the extended time-domain signal and envelope spectrum. Compared to before extension, the fault characteristic frequency and its harmonics remain consistent with no significant changes.

MOMEDA deconvolution inevitably causes time-domain truncation, leading to data loss and reduced frequency resolution. Therefore, a signal mirror extension strategy must be introduced for compensation. By comparing the envelope spectra before and after extension in Figure 12 and Figure 13, it can be seen that while this strategy perfectly restores the time-domain waveform, the fundamental fault frequency and its harmonics remain highly consistent with those before extension. This fully demonstrates its effectiveness in losslessly compensating for signal length and retaining weak impact features with high fidelity.

## 4. Experimental Verification

**Figure 14:**
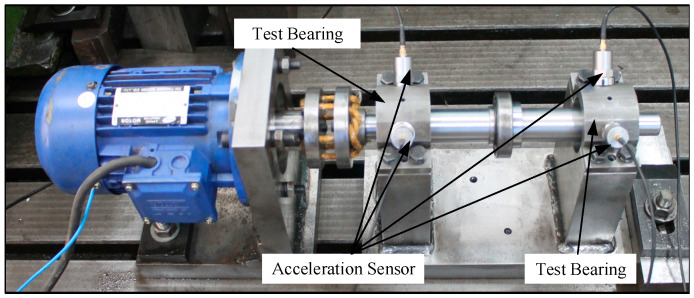
Test Rig for Fault Vibration Signal of Rolling Bearings.

Figure 14 shows the test rig for the vibration signal of the rolling bearing fault. To comprehensively capture the multi-directional vibration responses of the bearing, four CT1050LC piezoelectric accelerometers are employed in the experiment. They are symmetrically arranged at both ends of the bearing housing, with an installation angle of 45° relative to the radial direction of the rotor. The bearings at both ends of the test rig are faulty 6206 bearings, and their parameters are listed in Table 3. In the experiment, the bearing speed is about 1480 rpm, and the sampling frequency is 10 kHz. The fault characteristic frequencies are *f_out_* = 88.01 Hz, *f_in_* = 134.00 Hz, *f_ball_* = 115.50 Hz.

**Figure 15:**
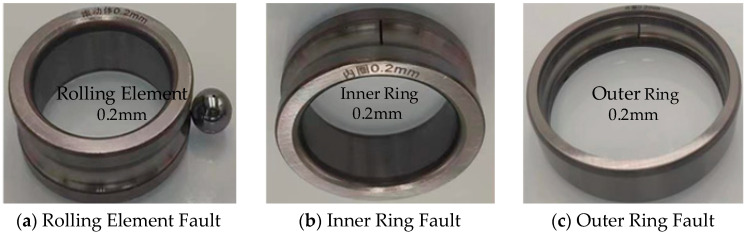
The fault width of the rolling bearing is 0.2 mm.

The faults in the test bearings are artificially induced by creating local rectangular groove defects using electrical discharge machining. When a rolling element passes over this material-loss zone, the abrupt change in contact status excites periodic impact vibrations. Depending on the fault location, the corresponding impact periods vary, which are ultimately mapped as specific fundamental fault characteristic frequencies and their harmonic components in the envelope spectrum. Bearings with fault widths of 0.2 mm on the inner race, outer race, and rolling element, as shown in Figure 15, are selected for the experiment.

### 4.1. Inner Race Fault

**Figure 16:**
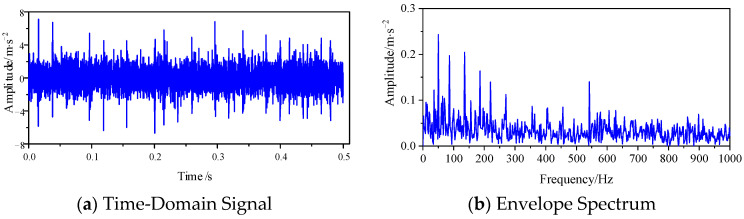
Time-domain signal and envelope spectrum of inner ring fault of rolling bearing.

The time-domain signal and envelope spectrum of the rolling bearing inner race fault signal are shown in Figure 16. Due to noise interference and the weak fault signal, it is difficult to identify the fault features in the envelope spectrum.

**Figure 17:**
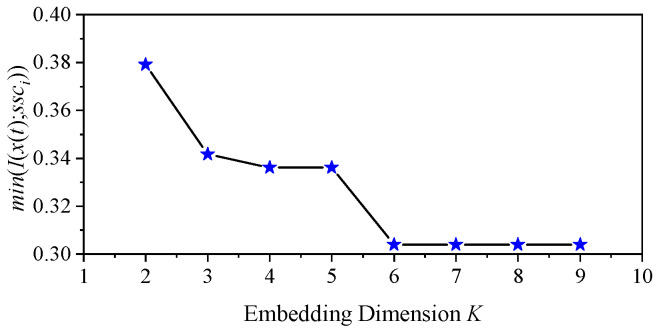
Determine the optimal embedding dimension *K* for the inner race fault signal.

First, the mutual information between SSC*_i_* and the original vibration signal *x*(*t*) is calculated as $\left\{I(x(t);SSC_{i}),i=1,2,3\ldots K\right\}$. The optimal embedding dimension K is determined to be 6 based on Figure 17.

**Figure 18:**
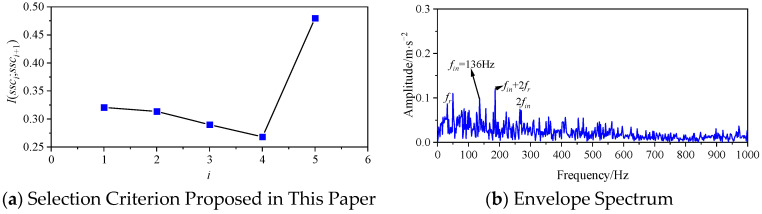
The selection criterion and its envelope spectrum proposed in this paper.

Then, the $I(SSC_{i};SSC_{i+1})$ values of each component are calculated as shown in Figure 18a, where a sudden change occurs at *i* = 4 and $I(SSC_{3};SSC_{4})$ is less than $I(SSC_{5};SSC_{6})$. Therefore, SSC_4_ is determined to be the optimal component. As can be seen from Figure 18b, a frequency peak of 136 Hz is identified, which is close to the inner ring fault characteristic frequency. However, other harmonics are not prominent. Additionally, sidebands with rotational frequency 2*f_r_* appear on both sides of the fault frequency *f_i_*, indicating that the fault characteristic frequency *f_in_* is modulated by the rotational frequency *f_r_*.

**Figure 19:**
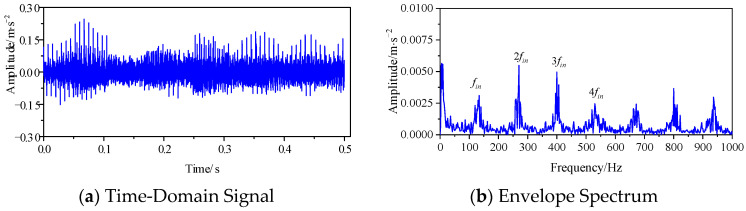
MOMEDA signal processing of the inner race fault (0.2 mm).

MOMEDA is applied to process the optimal component SSC_4_. Based on the fault frequency identified in Figure 18, the convolution period is set to 74, and the filter length is 600. Figure 19 shows the time-domain signal and envelope spectrum after waveform extension. After waveform extension, the signal clearly identifies the inner race fault characteristic frequency and its harmonics.

**Figure 20:**
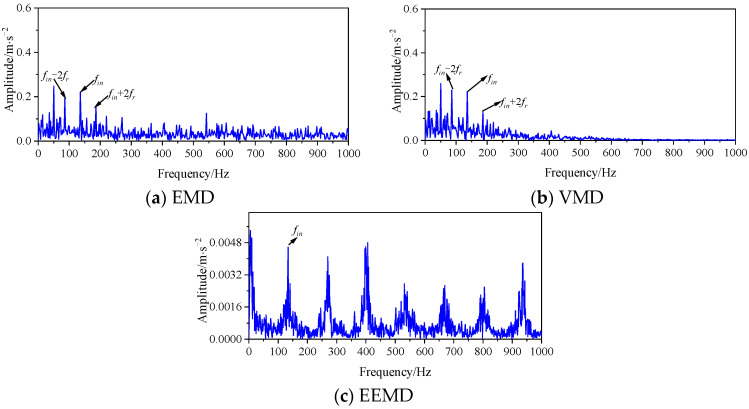
The envelope spectrum of EMD, VMD and EEMD.

To demonstrate the superiority of the method proposed in this paper, the EMD and VMD methods are used to decompose the original signal. Envelope spectrum analysis is then performed on the optimal components of the decomposed signals. The results are shown in Figure 20. Although both methods can identify the fault frequency, the fault frequency is interfered with by other frequencies, and the optimal component from the VMD method shows a missing fault frequency band. For further comparative analysis, the original inner race fault signal is subjected to EEMD [16] decomposition. Although the reconstructed signal after decomposition can identify the inner race fault frequency and its first four harmonics, the higher-order harmonic features are relatively blurred, and both the spectral line continuity and the degree of feature prominence remain insufficient.

**Figure 21:**
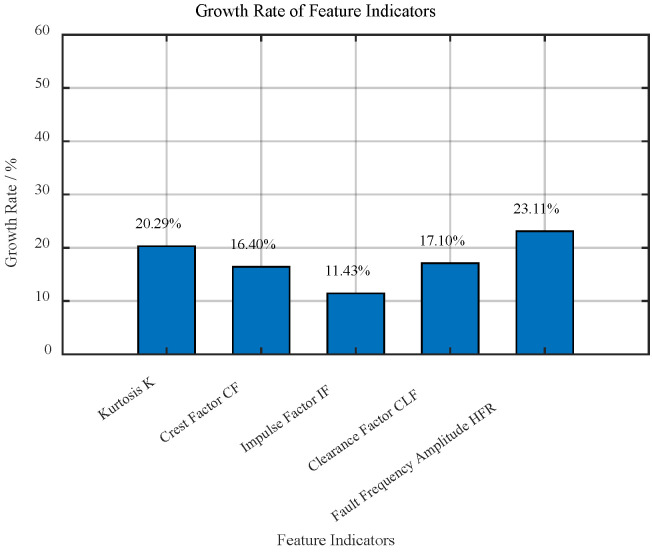
Improvement rates of various indicators for the inner race fault signal after MOMEDA processing.

A quantitative analysis is performed on the inner race fault signal, and the results are shown in Table 4 and Figure 21. It can be observed that the various characterization parameters are improved to varying degrees, with increases ranging from 11.43% to 23.11%.

### 4.2. Outer Race Fault

**Figure 22:**
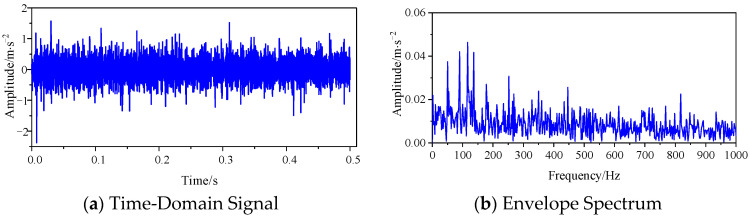
Time-domain signal and envelope spectrum of outer ring fault of rolling bearing.

Figure 22 shows the time-domain signal and envelope spectrum of the rolling bearing outer race fault. Due to noise interference, the fault characteristic frequency is not prominent in the envelope spectrum, so the method proposed in this paper is applied to process the signal.

**Figure 23:**
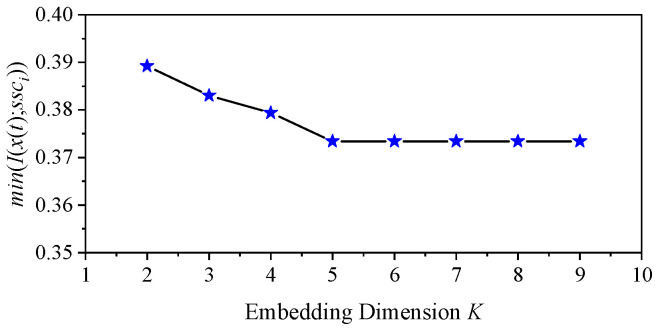
Determine the optimal embedding dimension *K* for the outer race fault signal.

First, the mutual information between SSC*_i_* and the original vibration signal *x*(*t*) is calculated as $\left\{I(x(t);SSC_{i}),i=1,2,3\ldots K\right\}$. The optimal embedding dimension *K* is determined to be 5 based on Figure 23.

**Figure 24:**
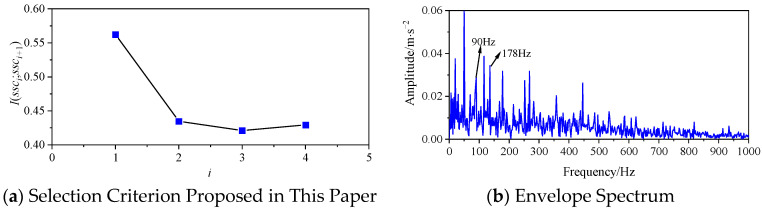
The determination of the optimal component and its envelope spectrum for the outer race fault signal.

Then, the $I(SSC_{i};SSC_{i+1})$ values of each component are calculated as shown in Figure 24. A sudden change occurs at *i* = 3 and $I(SSC_{4};SSC_{5})$ is less than $I(SSC_{2};SSC_{3})$. Therefore, SSC_4_ is chosen as the optimal component. As can be seen from Figure 24, significant frequency peaks appear at 90 Hz and 178 Hz, which are close to the outer ring fault characteristic frequency and its second harmonic. However, other harmonics are not prominent.

**Figure 25:**
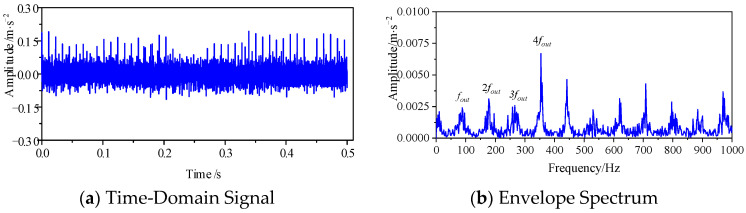
MOMEDA Signal Processing of the outer race fault (0.2 mm).

MOMEDA is applied to process the optimal component SSC_4_. Based on the fault frequency identified in Figure 24, the convolution period is set to 114, and the filter length is 600. Figure 25 shows the time-domain signal and envelope spectrum after waveform extension. After waveform extension, the signal clearly identifies the outer race fault characteristic frequency and its harmonics.

**Figure 26:**
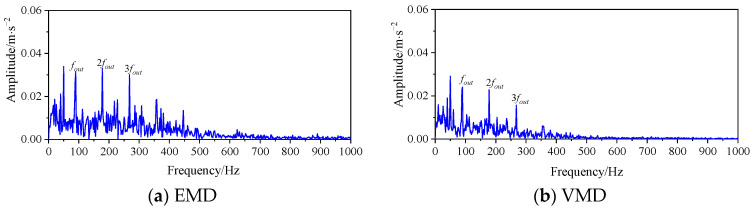
The envelope spectrum of EMD and VMD for the outer race fault signal.

To demonstrate the superiority of the method proposed in this paper, the EMD and VMD methods are used to decompose the original signal. Envelope spectrum analysis is then performed on the optimal components of the decomposed signals. The results are shown in Figure 26. The envelope spectrum of the optimal components from EMD and VMD can only identify part of the fault characteristic frequencies, and there are missing fault frequency bands.

**Figure 27:**
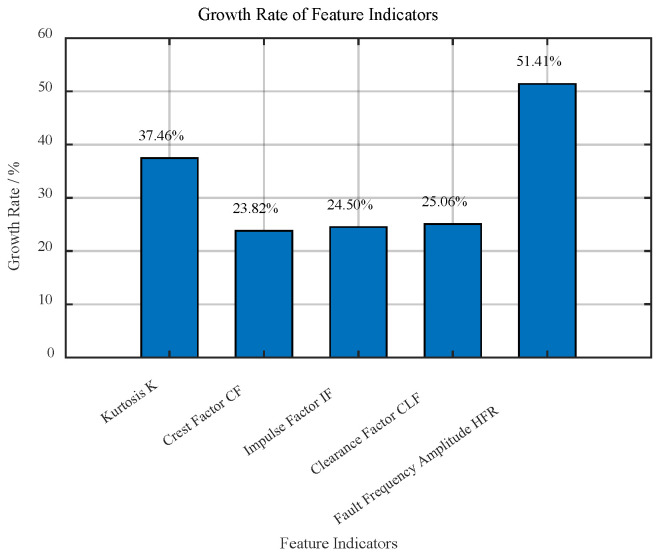
Improvement rates of various indicators for the outer race fault signal after MOMEDA processing.

A quantitative analysis is performed on the outer race fault signal, and the results are shown in Table 5 and Figure 27. It can be observed that the various characterization parameters are improved to varying degrees, with increases ranging from 23.82% to 51.41%.

### 4.3. Rolling Element Fault

**Figure 28:**
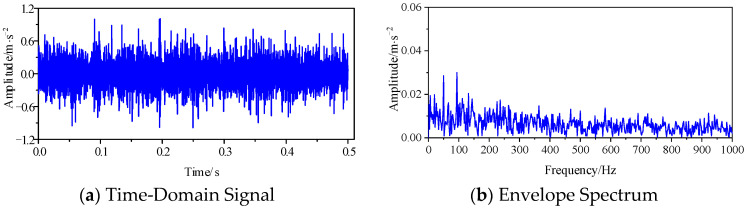
Time domain and envelope spectrum of rolling element fault of rolling bearing.

The time-domain waveform and envelope spectrum of the rolling element fault in the rolling bearing are shown in Figure 28. Due to noise interference and the weak fault signal, the fault characteristic frequency is not clearly visible in the envelope spectrum, so the method proposed in this paper is applied to process the original fault signal.

**Figure 29:**
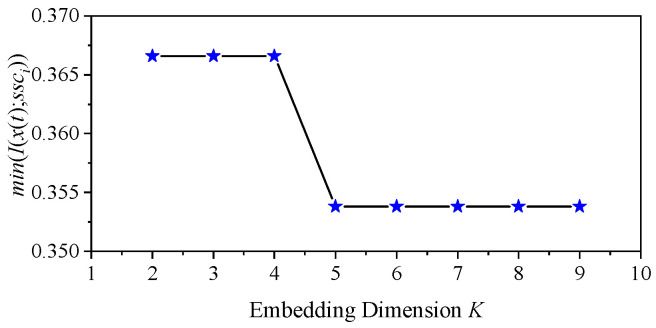
Determine the optimal embedding dimension *K* for the rolling element fault signal.

First, the mutual information between SSC*_i_* and the original vibration signal *x*(*t*) is calculated as $\left\{I(x(t);SSC_{i}),i=1,2,3\ldots K\right\}$. The optimal embedding dimension *K* is determined to be 5 based on Figure 29.

**Figure 30:**
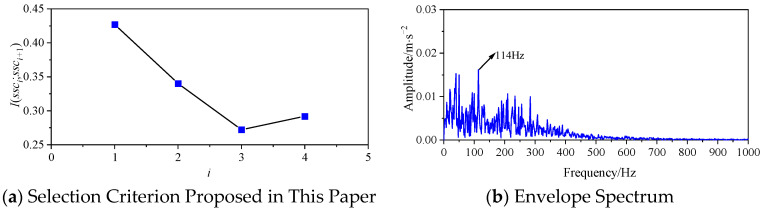
The determination of the optimal component and its envelope spectrum for the rolling element fault signal.

Then, the $I(SSC_{i};SSC_{i+1})$ values of each component are calculated. The calculation results are shown in Figure 30. A sudden change occurs at *i* = 3 and $I(SSC_{3};SSC_{4})$ is the smallest. Therefore, SSC_4_ is chosen as the optimal component. Envelope analysis is performed on it as shown in Figure 30. A frequency peak of 114 Hz can be seen, which is close to the rolling element fault characteristic frequency. However, other harmonics are not prominent.

**Figure 31:**
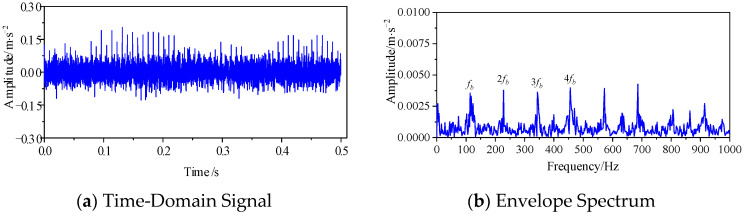
MOMEDA signal processing of the rolling element fault (0.2 mm).

MOMEDA is applied to process the optimal component SSC_4_. Based on the fault frequency identified in Figure 30, the convolution period is set to 88, and the filter length is 600. Figure 31 shows the time-domain signal and envelope spectrum after waveform extension. After waveform extension, the signal clearly exhibits fault impacts and periodicity. The fault frequency in the envelope spectrum is prominent and can effectively identify the rolling element fault characteristic frequency and its harmonics.

**Figure 32:**
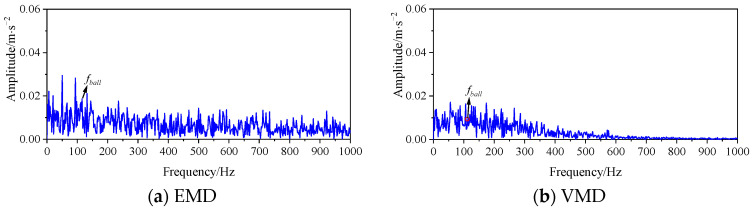
The envelope spectrum of EMD and VMD for the rolling element fault signal.

To demonstrate the superiority of the method proposed in this paper, the EMD and VMD methods are used to decompose the original signal. Envelope spectrum analysis is then performed on the optimal components of the decomposed signals. The results are shown in Figure 32. After decomposition with EMD and VMD, the fault characteristic frequency cannot be identified, and, thus, the fault type of the rolling bearing cannot be determined.

**Figure 33:**
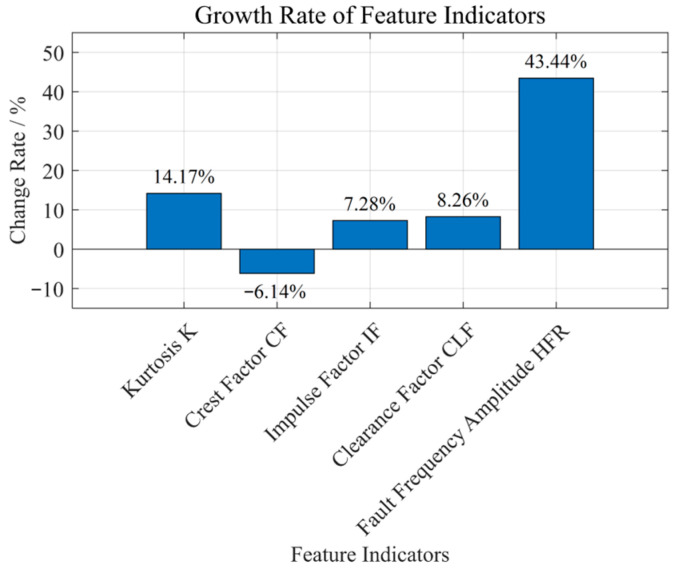
Improvement rates of various indicators for the rolling element fault signal after MOMEDA processing.

A quantitative analysis is performed on the rolling element fault signal, and the results are shown in Table 6 and Figure 33. It can be observed that, except for a slight decrease in the crest factor (6.1%), the other characterization parameters are improved to varying degrees, with increases ranging from 7.28% to 43.44%.

### 4.4. Comparative Analysis of Different Fault Sizes

**Figure 34:**
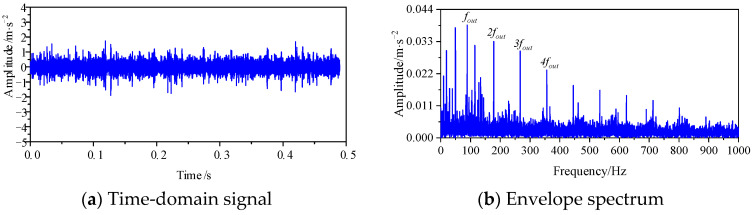
Time-domain signal and envelope spectrum of the 0.6 mm outer race fault in the rolling bearing.

For the experimental data with an outer race fault size of 0.6 mm in the bearing dataset, the outer race fault frequency of the test bearing is 89 Hz. Figure 34 shows the time-domain signal and envelope spectrum of the 0.6 mm outer race fault signal from the rolling bearing. Due to noise interference, the fault characteristic frequency is not prominent in the envelope spectrum.

**Figure 35:**
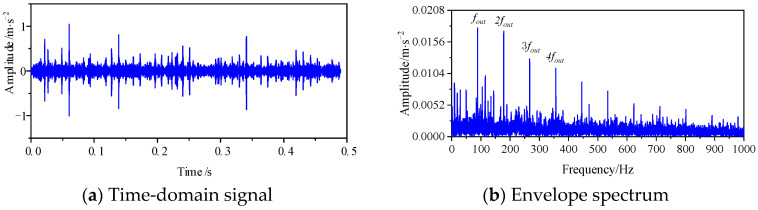
MOMEDA signal processing of the outer race fault (0.6 mm).

Based on the method proposed in Section 4.2, the optimal embedding dimension *K* is determined to be 5. The *I*(SSC*_i_*; SSC*_i_*_+1_) values of each component are calculated as shown in Figure 24. A sudden change occurs at *i* = 3, and *I*(SSC_4_; SSC_5_) is less than *I*(SSC_2_; SSC_3_). Therefore, SSC_4_ is chosen as the optimal component. MOMEDA is applied to process the optimal component SSC_4_. Based on the identified fault frequency, the convolution period is calculated as 114, and the filter length is 600. Figure 35 shows the time-domain signal waveform and envelope spectrum after OSSD. From the envelope spectrum, the outer race fault characteristic frequency and its harmonics can be identified.

By comparing and analyzing Figure 25 and Figure 35, it can be observed that for faults of different sizes, the method proposed in this paper can effectively identify the fault characteristic frequency and its harmonics, indicating that the method possesses good robustness.

## 5. Conclusions

An early fault feature extraction method for rolling bearings combining OSSD and MOMEDA is proposed. This method effectively decomposes non-stationary vibration signals using OSSD. While adaptively resolving the mode mixing issue, it introduces mutual information criteria to achieve the autonomous selection of optimal components, overcoming the reliance on manual prior experience in traditional SSD algorithms.

The evolution process of MOMEDA in enhancing weak impact features is elucidated, and a signal mirror extension strategy is introduced to address the inherent signal length reduction and endpoint effects caused by deconvolution. This strategy successfully compensates for missing data in the time domain, ensures the integrity of the full-length signal, and achieves high-fidelity waveform reconstruction.

Simulation and bearing experimental results demonstrate that the proposed method possesses extremely high feature recognition accuracy and robustness in a strong background noise environment. It is significantly superior to traditional EMD and VMD methods in terms of the clarity and completeness of the fault frequency and its higher-order harmonics.

The proposed method exhibits strong robustness for both noise-added simulation signals and test signals from our specific experiments. Through adaptive optimal selection and denoising by OSSD, combined with MOMEDA utilizing the multipoint D-norm to force the inband periodic impacts to superimpose in phase, the method consistently maintains high-fidelity and stable extraction of weak fault features.

## Figures and Tables

**Table 1:** Parameter setting of simulation signal.

*A*_0_	*f_r_*	*C*	*f_n_*	*f_i_*	Signal-to-Noise Ratio
0.35	30	650	4000	120	−20 dB

**Table 2:** Quantitative comparison of the simulation signal before and after processing.

Data Type	Kurtosis	Crest Factor	Impulse Factor	Margin Factor	Fault Harmonic Energy Ratio in Envelope Spectrum
Simulation Signal Before Decomposition	3.0005	3.3906	4.2569	5.0295	0.11065
Optimal Component SSC_3_	4.1225	3.7825	4.7771	5.6688	0.16281

**Table 3:** Rolling Bearing Parameters.

Bearing Type	Number of Rolling Elements	Rolling Element Diameter	Pitch Circle Diameter	Contact Angle
6206	9	9.525 mm	46 mm	0°

**Table 4:** Quantitative comparison of the inner race signal before and after processing.

Data Type	Kurtosis	Crest Factor	Impulse Factor	Margin Factor	Fault Harmonic Energy Ratio in Envelope Spectrum
Simulation Signal Before Decomposition	24.163	7.7722	16.531	14.354	0.33072
Component SSC_4_ After MOMEDA Processing	29.066	9.0469	18.421	16.808	0.40716

**Table 5:** Quantitative comparison of the outer race signal before and after processing.

Data Type	Kurtosis	Crest Factor	Impulse Factor	Margin Factor	Fault Harmonic Energy Ratio in Envelope Spectrum
Simulation Signal Before Decomposition	2.5476	4.1781	5.2999	6.2917	0.35559
Component SSC_4_ After MOMEDA Processing	3.5019	5.1733	6.5983	7.8685	0.35559

**Table 6:** Quantitative comparison of the rolling element signal before and after processing.

Data Type	Kurtosis	Crest Factor	Impulse Factor	Margin Factor	Fault Harmonic Energy Ratio in Envelope Spectrum
Simulation Signal Before Decomposition	3.8659	5.7687	6.8544	8.0844	0.42323
Component SSC_4_ After MOMEDA Processing	4.4137	5.4168	7.3533	8.7523	0.60708

## Data Availability

The original contributions presented in this study are included in the article. Further inquiries can be directed to the corresponding author.

## Abbreviations

The following abbreviations are used in this manuscript:

OSSD	Ordered Singular Spectrum Decomposition
MOMEDA	Multipoint Optimal Minimum Entropy Deconvolution Adjusted
EMD	applied the empirical mode decomposition
IMF	intrinsic mode function
SVDD	support vector data description
VMD	Variational Modal Decomposition
ChOA	Chimp Optimization Algorithm
EEMD	ensemble empirical mode decomposition
MCTFTSR	multi-parameter constrained time-delay feedback tri-stable stochastic resonance
SSD	singular spectrum decomposition
CNN	convolutional neural network
DWT	discrete wavelet transform
LSTMs	long short-term memory networks
